# Internal medicine residents’ achievement goals and efficacy, emotions, and assessments

**Published:** 2018-11-12

**Authors:** Lia Daniels, Vijay Daniels

**Affiliations:** 1 Department of Educational Psychology, Faculty of Education, University of Alberta, Alberta, Canada; 2 Department of Medicine, Faculty of Medicine and Dentistry, University of Alberta, Alberta, Canada

## Abstract

**Background:**

Achievement goal theory is consistently associated with specific cognitions, emotions, and behaviours that support learning in many domains, but has not been examined in postgraduate medical education. The purpose of this research was to examine internal medicine residents’ achievement goals, and how these relate to their sense of self-efficacy, epistemic emotions, and valuing of formative compared to summative assessments. These outcomes will be important as programs transition more to competency based education that is characterized by ongoing formative assessments.

**Methods:**

Using a correlational design, we distributed a self-report questionnaire containing 49 items measuring achievement goals, self-efficacy, emotions, and response to assessments to internal medicine residents. We used Pearson correlations to examine associations between all variables.

**Results:**

Mastery-approach goals were positively associated with self-efficacy and curiosity and negatively correlated with frustration and anxiety. Mastery-approach goals were associated with a greater value for feedback derived from annual ACP exams, end-of-rotation written exams, and annual OSCEs. Performance-approach goals were only associated with valuing ACP exams.

**Conclusion:**

Mastery-approach goals were associated with self-efficacy and epistemic emotions among residents, two constructs that facilitate autonomous learning. Residents with mastery-approach goals also appeared to value a wider range of types of assessment data. This profile will likely be beneficial for learners in a competency-based environment that involves high levels of formative feedback.

## Introduction

**Figure 1:**
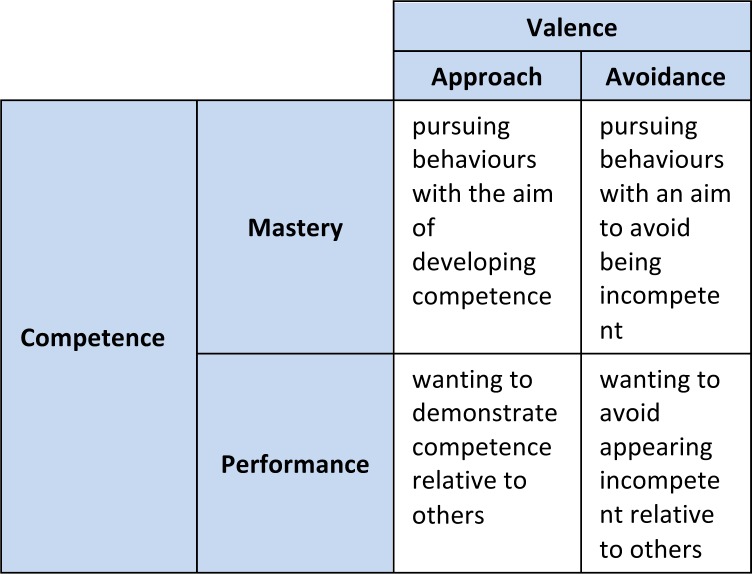
2 x 2 model of achievement goals and definitions

Motivation is broadly defined as the process by which goal-directed behaviour is initiated and sustained. Recently Cook and Artino^1^ argued the importance of “mak[ing] the theoretical foundations of motivation accessible to medical educators” (p. 998) and they included achievement goal theory as one of five contemporary theories of motivation that may be highly relevant to medical education. Medical education, including postgraduate residency programs, represents a competitive achievement context in which individuals exert effort to obtain desirable outcomes. Achievement goal theory uses the term “goal” to represent different aims a learner has in the particular achievement context.^2,3^ Specifically, in the hierarchical model of achievement goals, goals are conceptualized as consisting of two dimensions. The first dimension is competence and represents the extent to which individuals strive to gain competence or demonstrate competence. The former is labeled a mastery goal and the latter is labeled a performance goal. The second dimension is valence and represents the extent to which individuals move toward or away from their goal (approach or avoidance goals, respectively). Crossing competence and valence results in a 2 x 2 matrix with four types of goals: mastery-approach, mastery-avoidance, performance-approach, and performance-avoidance (Figure 1). Each goal is associated with a relatively unique pattern of cognitions, emotions, and behaviours that can support or impede student learning and success.^4^

The relevance of achievement goals in academic (K-12 and post-secondary), sport, and work domains is well established because approach goals are more adaptive than avoidance goals.^5^ Mastery-approach goals are often considered the most beneficial of the four types of goals showing positive associations with persistence, self-regulated learning, positive emotions, and effort, although not necessarily academic grades.^6-10^ In contrast, both mastery-avoidance and performance-avoidance goals tend to be associated with maladaptive outcomes including heightened anxiety, poor grades, and low self-regulation.^11,12^ Performance-approach goals have the most complex pattern of outcomes often related to higher grades but also higher anxiety and procrastination, and lower perceptions of success and cognitive processing.^6,13^ Both cognitive and environmental interventions can help students embrace mastery-approach goals and reap the associated benefits.^14,15^

Some research has focused on achievement goals specifically in medical students, and again mastery-approach emerges as the most adaptive. Mastery-approach goals correlated positively with how much medical students monitored their studying, used a deep approach to studying, and organized their studying^16^ and helped students see their curriculum as providing opportunities to develop rather than simply demonstrate competence.^17^ Performance-approach goals were positively correlated with organization of learning only.^16^ Achievement goals of medical students who recently entered clerkship did not have a direct relationship with either preceptor evaluations or pass/fail on a standardized-patient exam, but mastery-approach goals did.^17^ Both mastery-avoidance and performance-avoidance goals were positively correlated with surface approach to learning reinforcing that these are generally maladaptive goals.^16^

Research on achievement goals in postgraduate medical education is sparse leaving important questions unanswered. For example, what are the relative levels of achievement goals for residents? As a group are they more likely to report mastery-approach goals as they strive to become competent professionals or do they report performance-approach goals in a system that remains competitive? How common are mastery- and performance-avoidance goals during this period of skill accumulation? Moreover, there is no understanding of the relationships amongst residents’ achievement goals and certain cognitions, emotions, and assessments that are commonly associated with success. Toward this end, we have chosen to focus on self-efficacy, epistemic emotions, and different types of summative and formative assessment information as outcomes supportive of success.

Self-efficacy is defined as “people’s judgments of their capabilities to organize and execute courses of action required to attain designated types of performances.”^18^ Epistemic emotions are defined as emotions such as surprise or confusion that arise in response to the qualities of a learning task.^19^ Finally, summative and formative assessments are defined by the purpose of the assessment.^20^ The purpose of summative assessment is quality control: to assess student learning at the end of a unit. The purpose of formative assessment is learning: to use ongoing assessment data to support student progress and refine instruction. Feedback, reflection, and portfolios are common types of formative assessment. In particular, there is a substantial amount of evidence supporting the important role of feedback in student learning.^20^ We argue that residents’ perceptions of self-efficacy, epistemic emotions, and their valuing of formative over summative types of assessment are three areas that may be strongly related to their achievement goals as have been shown in other samples of students.^21-23^

### Efficacy, emotions, and assessment: hallmarks of adaptive motivation

Self-efficacy is arguably the most important cognition related to student success. Self-efficacy has proven to be the strongest psychosocial predictor of undergraduate college students’ achievement and persistence with a larger effect size than Standardized Achievement Test scores.^24,25^ In medical school specifically, researchers examined students’ self-efficacy in three domains of problem-based learning (PBL): group interactions, problem solving, and responsibility for learning. Each sub-domain of self-efficacy was positively correlated with students’ motivation to learn, planning and goal setting, strategy use and assessment, and lack of self-directedness.^26^ One explanation for these associations is that self-efficacy is supported by four factors that may be high in PBL programs including mastery experiences, vicarious experiences, verbal persuasion, and emotional/physiological states.^27^ Outside of PBL, mastery-approach goals are regularly positively associated with self-efficacy.^22^ If mastery-approach goals are one route to enhance efficacy, then understanding this relationship in postgraduate trainees is crucial.

The study of students’ achievement emotions has been rapidly increasing in general student populations and revealing important implications for students’ learning.^28^ Achievement emotions can be elicited by students’ cognitive appraisals related to their sense of control and value over their learning activities and outcomes. High appraisals of control and value tend to be associated with pleasant emotions such as enjoyment or hopefulness whereas low control and value appraisals tend to be associated with unpleasant emotions such as boredom or hopelessness. In one study with medical students, Artino and colleagues showed that students’ emotions explained 14% of their national board shelf examination scores and 20% of their course grade.^29^ Other than this study, however, emotions remain underrepresented in research in medical education.^30^ This omission is particularly noticeable for epistemic emotions such as surprise, curiosity, and confusion, which are “triggered by the cognitive characteristics of tasks [and] can be of fundamental importance for learning.”^19^ No research has considered residents’ epistemic emotions even though they may regularly experience epistemic emotions as they seek out information related to patient cases. For example, residents may find a surprising contraindication, or this same contraindication may lead to frustration or even anxiety. Moreover, because achievement goals have been regularly associated with achievement emotions^23^ understanding how goals support or hinder epistemic emotions is important.^19^

Finally, in both compulsory schooling^31^ and postgraduate medical education,^30^ there is a shifting emphasis from summative assessments *of* learning to formative assessments *for* learning. Assessment for learning is one of the core assessment principles in competency-based medical education,^32^ a growing and important movement globally but especially in Canada.^33^ In an assessment for learning environment, rather than being a marker of learning at the end of block, rotation, or program, assessment data is viewed as a source of information to guide residents’ educational path. Although few have investigated relationships between specific types of assessment (see Pekrun and colleagues^34^ as an exception), based on the theoretical underpinnings it seems reasonable to suggest that motivation is related to different types of assessments. Performance-approach goals likely orient students to traditional summative assessments and mastery-approach goals likely orient students to contemporary formative assessments.^35^ In residents, there is some qualitative evidence that they struggle to see the value of assessment for learning, and indeed their personal goals may shape how much they value different types of assessment.^36^ This relationship, however, has not been explored empirically.

### The purpose of this study

We decided to explore postgraduate resident trainees’ levels of achievement goals or their relationships with self-efficacy, epistemic emotions, and valuing of various program assessments. We hypothesized that residents would most strongly report mastery-approach goals and that mastery-approach would be associated with more self-efficacy, adaptive epistemic emotions, and increased value for various forms of assessment feedback.

## Methods

### Participants and procedures

We used a correlational self-report design to collect data from 99 postgraduate year (PGY) 1-3 internal medicine residents at the University of Alberta in Edmonton, Alberta, Canada. A research assistant attended an academic half-day and distributed paper copies of the questionnaire for residents to complete onsite. Participation was voluntary and took approximately 20 minutes of the session. Two procedures were in place to reduce feelings of coercion/pressure. First, everyone in attendance received a questionnaire regardless of whether or not they wanted to participate and were encouraged to simply hand in a blank form if they chose not to participate. Second, there were no administrators or faculty members in the room during the data collection. To include residents who were unable to attend the half-day we circulated the questionnaire via email. All participants received a $5 gift card to Starbucks as a thank you from the researchers. These procedures were approved by the University of Alberta’s Research Ethics Board 2.

### Measures

In addition to basic demographic information (age, gender, and year in residency), the questionnaire measured achievement goals (16 items), self-efficacy (3 items), epistemic emotions (12 items), and valuing of assessments used in the program (13 items). All non-assessment-related questionnaire items were based on pre-existing scales which we modified by changing “school” or “work” statements in the items or instructions to statements more specific to “residency” in order to increase the domain specificity of the scale.

For achievement goals, we used a shortened version of Baranik et al.’s^37^ 2 x 2 measure of achievement goals for the work domain, which had evidence of adequate reliability and convergent and divergent validity with employed psychology undergraduate students in two studies (coefficient alpha range .69 to .82). We reduced the 11 mastery-avoidance items to four items to match the number of items in the other three domains and to reduce participant fatigue in answering the questionnaire. We did not run a pilot study on the reduced four items. We were relatively confident that four items would be adequate to measure the construct because many other achievement goal measures with adequate evidence of reliability and validity require only three or four items per subscale.^38^ Participants responded to the prompt “In residency, my goal is…” on a 1 to 7 rating scale anchored by “strongly disagree” and “strongly agree.”

We used three items to measure residents’ self-efficacy for their residency program. The three items were based on recommendations from Klassen and Durksen^39^ when seeking a very short measure. Although the three-item scale was originally used with pre-service teachers, it was employed during a study on practicum placements, which might be considered similar to residency training. Their study provided evidence of adequate reliability over eight time points (coefficient alpha ranged from .79 to .94). Participants responded on a four-point rating scale where 1 = almost never, 2 = sometimes, 3 = often, and 4 = always as recommended by Schwarzer and Jerusalem.^40^

Following Pekrun and colleagues, we collected data on 12 epistemic emotions in four categories.^19^ Their original validation paper suggested three items per category provided excellent evidence of reliability and validity. High valence positive activating emotions were measured by surprise, excited, astonished (labeled surprise) whereas moderate valence positive activating emotions were measured by curious, interested, and inquisitive (labeled curiosity). Moderate valence negative activating emotions were measured by frustrated, muddled, and irritated (labeled frustrated); whereas, high valence negative activating emotions were measured by nervous, worried, and anxious (labeled anxiety). Participants were asked to “think of a specific case you recently read around” and indicate the extent to which they experienced each emotion on a 1 to 5 rating scale where 1 = not at all, 2 = very little, 3 = moderate, 4 = a little, and 5 = very much.

The associate program director for assessment identified five categories of assessments that could support residents’ goals: 1) studying for and feedback from the annual American College of Physicians (ACP) Exam, 2) studying for and feedback from end-of-rotation written examinations, 3) studying for and feedback from the annual Objectives Structured Clinical Examination (OSCE), 4) workplace-based observation and feedback, and 5) reflection activities. The first three types of assessments represent traditional summative assessments of learning and thus may be preferable by students with performance-approach goals who look to show their competence relative to others. The last two types of assessment represent formative assessments of learning that may be more appealing for students with mastery-approach goals who desire to increase their competence on an intra-individual level. Participants responded to the extent to which these different assessments supported their goals on a 1 to 7 rating scale anchored by “not at all” and “very much”.

### Analysis

All scales were summed such that higher scores represent a stronger identification with the underlying construct. We computed a coefficient alpha as a measure of reliability for each subscale and examined the descriptive statistics for all measures. We used SPSS^41^ for the main correlational analyses between all aforementioned variables.

## Results

Fifty-one of approximately 60-70 residents in attendance at half-day completed the questionnaire with an additional 16 residents completing it online. The mean age was 28 years old, 58% were male and 42% female (none chose the non-binary gender classification), and 37% were PGY-1, 35% PGY-2, and 28% PGY-3.

The scales for mastery-approach, performance-approach, and performance-avoidance had acceptable alpha reliabilities (0.76-0.88). However, the reliability for mastery-avoidance (the domain that was shortened) was unacceptable and therefore omitted from all correlational analyses. All other measures had acceptable reliabilities between 0.61-0.93. Residents reported mastery-approach goals more than a full point above the next highest reported goal, which was performance-approach (Table 1).

For the main analyses, within the goals measures, mastery-approach goals were negatively related to performance-avoidance goals and unrelated to performance-approach; whereas, performance-approach and performance-avoidance were positively correlated (Table 2). Within the epistemic emotions, several expected correlations occurred; surprise and curiosity were positively correlated with each other, anxiety and frustration were positively correlated with each other, and frustration and curiosity were negatively correlated with each other. These appropriate relationships provide some evidence of validity for the constructs.

**Table 1:** Descriptive statistics for all study variables

	Sample item	N items	*M*	SD	Range	Skewness	
**Goals (7-point scale)**							
Mastery-approach	For me, development of my skills is important enough to take risks	4	5.70	1.05	2.25 - 7.00	-1.09	.87
Mastery-avoidance	I just try to avoid being incompetent at performing the skills and tasks necessary in residency.	4	3.90	1.15	1.00 - 6.50	-.23	.47
Performance-approach	I prefer to work on cases where I can prove my ability to others.	4	4.48	1.30	2.00 - 7.00	-.05	.76
Performance-avoidance	I would avoid taking on a new task if there was a chance that I would appear incompetent to others.	4	3.05	1.39	1.00 - 7.00	.39	.88
**Self-Efficacy (4-point scale)**							
Self-efficacy	I’m confident I can do an excellent job in residency.	3	3.08	.75	1.33 - 4.00	-.18	.93
**Emotions (5-point scale)**							
Surprise	Surprise	3	2.50	.93	1.00 - 5.00	.45	.75
Curiosity	Curiosity	3	4.09	.66	2.00 - 5.00	-.76	.61
Frustration	Frustration	3	1.70	.83	1.00 - 4.33	1.34	.77
Anxiety	Anxiety	3	1.79	.87	1.00 - 4.33	1.11	.84
**Formal and Informal Assessments (7-point scale)**							
ACP examination	Finding out in which areas you struggled on the annual ACP exam.	3	4.16	1.63	1.00 - 7.00	-.31	.87
End-of-rotation written examinations	Studying for and writing end of rotation exams.	2	4.66	1.58	1.00 - 7.00	-.55	.72
OSCEs	Studying for and taking the annual OSCE.	4	5.21	1.11	1.50 - 7.00	-.92	.81
Workplace-based observation and feedback	Getting feedback on your diagnosis and management after reviewing a case with the fellow or attending.	2	5.61	1.31	2.00 - 7.00	-.94	.66
Reflection activities	Reflecting on each rotation by writing a learning plan.	2	3.56	1.51	1.00 - 7.00	-.09	.82

**Table 2:** Zero-order correlations between all study variables

	1	2	3	4	5	6	7	8	9	10	11	12
**1. Mastery-approach**												
**2. Performance-app.**	.08											
**3. Performance-avoid.**	-.46^**^	.28^*^										
**4. Self-efficacy**	.51^**^	.05	-.39^**^									
**5. Surprise**	.09	.10	-.10	-.02								
**6. Curiosity**	.47^**^	.12	-.10	.47^**^	.35^**^							
**7. Frustrated**	-.40^**^	-.05	.34^**^	-.48^**^	.25	-.28^*^						
**8. Anxiety**	-.38^**^	.01	.31^*^	-.49^**^	.22	-.16	.67^**^					
**9. ACP**	.35^**^	.39^**^	-.12	.12	.09	.22	-.17	-.16				
**10. End-of-rotation exams**	.28^*^	.07	-.09	.01	.04	.11	.14	.06	.34^**^			
**11. OSCE**	.36^**^	.15	.21	.13	-.07	.22	-.05	-.01	.26^*^	.33^**^		
**12. Workplace feedback**	.22	.01	.01	.25^*^	-.13	.13	-.06	-.20	.10	.28^*^	.43^**^	
**13. Reflections**	.16	-.12	-.01	-.04	-.08	-.04	.16	.13	.13	.22	.39^**^	.41^**^

* *p* < .05

** *p* < .01

Next we consider the relationships between achievement goals, self-efficacy, epistemic emotions, and valuing summative and formative assessments. Mastery-approach goals were positively correlated with self-efficacy and with feeling curious, and negatively correlated with feeling anxious or frustrated during reading around important cases. Mastery-approach goals were also positively associated with valuing the ACP exam, the end-of-rotation written exams, and the annual OSCE as types of assessment that they view as supporting their goals. Finally, the performance goals had a few significant correlations. For example, performance-avoidance goals showed a negative correlation with self-efficacy and positive correlations with frustration and anxiety. For performance-approach goals the only significant correlation was with the ACP exam as an assessment that supports their goals.

## Discussion

The purpose of this research was to examine associations between achievement goals, self-efficacy, epistemic emotions, and internal medicine residents’ valuing of different summative and formative assessments. As this is one of the first investigations of achievement goals of internal medicine residents, we begin by discussing levels and relationships between the four goal types. Next, we focus on the adaptive associations that emerged for mastery-approach goals. Finally, we examine the less adaptive and narrow set of results for the performance-approach and avoidance goals.

In this sample, mastery-approach goals appear to be the best distinguished of the goals with a negative association with performance-avoidance goals and no significant correlation with performance-approach goals. Mastery-approach goals were also reported at a mean level more than a full point above the other three types of goals. Not only are these the most strongly reported goals, their associations with efficacy, emotions, and assessment are in line with research with other professional faculties such as education.^42^ Although most highly reported there remains room for improvement in terms of increasing mastery-approach and decreasing performance-avoidance. Working from the premise that scores of 5 or greater on the 7-point scale represent firm endorsement of the goal, then 23% of residents did not endorse mastery-approach goals and 14% endorsed performance-avoidance goals. Although cutting across valences is less theoretically grounded,^2^ the positive association between performance-approach and performance-avoidance may be related to an emerging argument that some learners see these as opposite ends of a single continuum in such a way that “doing better than others” is the same as “not doing worse than others.”^43^

Mastery-approach goals also had adaptive associations with self-efficacy and epistemic emotions. Specifically, residents who endorse mastery approach goals are more inclined to have positive appraisals of their self-efficacy suggesting that they are more confident in their abilities to meet the requirements of their training program. Mastery-approach goals were also negatively associated with anxiety and frustration and positively associated with curiosity. This suggests that when residents with mastery-approach goals read around their cases they are more likely to experience epistemic emotions that facilitate learning and less likely to experience emotions that hinder or impede learning.^19^

In contrast to mastery-approach goals, performance-avoidance goals were negatively related to assessments of self-efficacy, suggesting that performance-avoidance goals compromise confidence in abilities. Moreover, performance-avoidance goals were positively associated with experiencing the types of epistemic emotions that would undermine learning.

Neither Workplace-based observation with feedback nor Reflection activities were significantly correlated with a particular achievement goal, although the associations were in the positive direction with mastery-approach and may reach significance in a larger sample. Based on mean scores, residents valued workplace-based observation and feedback the most. The lack of correlation with achievement goal orientation could mean that this type of feedback on real-life performance is valued by all residents independent of goal orientation; i.e., if one is performance focused, feedback that one is doing well can be interpreted as better than others and not worse than others. In contrast, reflection with writing a learning plan was valued the least and also did not correlate with any goal orientation. Although we expected those who hold mastery-approach goals to value reflection, writing an action plan was a mandatory component of the reflection exercise which may undermine its value.

Residents who endorsed mastery-approach goals viewed feedback related to their ACP exam, end-of-rotation written examinations, and OSCEs as supporting their achieving mastery-approach goals. In contrast, residents who endorsed performance-approach goals viewed only the ACP exam as supporting their goals. This may be because that in addition to reporting areas for improvement by content domain, the ACP exam reports percentile scores (i.e., comparison to peers) whereas none of the other assessments do; thus matching the focus on normative achievement that is typical of performance-approach goals. Performance-avoidance goals did not correlate with perceptions of any type of assessments as supportive of their goals during training. In fact, these correlations were often near zero, suggesting that residents do not see any connection between the assessments and avoidance goals. This may make sense insomuch as avoidance goals are most concerned with avoiding negative outcomes and all assessments have the potential to impart negative information. Nonetheless, it reinforces that performance-avoidance goals do not position residents well for the transition to assessment *for* learning, such as competency-based medical education, which will involve more attention on incorporating all assessment data to guide their own further development.

### Implications for practice

As mentioned, 23% of the residents did not endorse mastery-approach goals and 14% endorsed performance-avoidance goals. If these numbers are similar in other residency programs we should consider encouraging mastery-approach goals in residency and minimizing performance-avoidance goals as much as possible. There are two major approaches to supporting mastery-goals, one focuses on changing the environment and the other on changing learners’ personal achievement goals. First, researchers have several recommendations for how to build a mastery-learning environment including allowing students to have choice, minimizing normative comparisons, and providing adequate time to meet program outcomes.^44^ Second, researchers have shown that brief psychosocial interventions such as attributional retraining^15,45^ can help students increase their endorsement of mastery-approach goals. Attributional retraining involves helping students shift their cognitions from uncontrollable and stable explanations for outcomes such as low ability to controllable and unstable explanations such as effort. In combination, environmental and individual interventions could be used to leverage mastery goals not only for postgraduate medical education but undergraduate as well.

### Limitations and future research

The results presented here need to be interpreted in light of the following three limitations. First, we did not undertake a formal validity study for any of the scales used even though this is their first use with internal medicine residents. Ideally all created and modified scales would have been subjected to a pilot study prior to investigating relationships. However, this procedure would have ultimately drained our pool of participants. Since the constructs are well established with evidence of reliability and validity in the broader literature there was little reason to suspect the scales would be particularly problematic. Indeed, the pattern of relationships between the constructs noted in the current study provides some evidence of validity. Second, although we represented 68% of the entire group of internal medicine residents, the sample was drawn from a single institution and was not sufficiently large to look for differences between years of training. Future research could be conducted at multiple institutions to increase the sample size and representativeness. Finally, all data collected were self-report, and future research should examine how achievement goals relate to more real-world indicators of clinical competence.

In conclusion, our results suggest that as is the case in many domains,^5^ mastery-approach goals appear to be the most common and most adaptive for internal medicine residents. Specifically, mastery-approach goals were associated with increased self-efficacy, curiosity in learning, and higher value on feedback from assessments, all of which may help residents thrive during the imminent shift to a competency-based medical education environment. Programs may want to measure examining residents’ goal orientation and consider environmental and individual interventions to support mastery-approach goals.

## References

[1] Cook DA, Artino AR Motivation to learn: an overview of contemporary theories. Medical education. 2016;50(10):997-1014. doi:10.1111/medu.1307 27628718 PMC5113774

[2] Elliot AJ Approach and avoidance motivation and achievement goals. Educational Psychologist. 1999;34(3):169-89. doi:10.1207/s15326985ep3403_3.

[3] Elliot AJ A conceptual history of the achievement goal construct In AJ. Elliot AJ & Dweck CS (eds.) Handbook of competence and motivation. New York, Guilford; 2005 p. 52-72.

[4] Elliot AJ, Dweck CS, Yeager DS, editors. Handbook of Competence and Motivation: Theory and Application Guilford Publications; 2017.

[5] Van Yperen NW, Blaga M, Postmes T A meta-analysis of self-reported achievement goals and nonself-report performance across three achievement domains (work, sports, and education). PloS ONE. 2014;9(4):e93594. doi: 10.1371/journal.pone.0093594. 24699695 PMC3974764

[6] Daniels LM, Haynes TL, Stupnisky RH, Perry RP, Newall NE, Pekrun R Individual differences in achievement goals: A longitudinal study of cognitive, emotional, and achievement outcomes. Contemporary Educational Psychology. 2008;33(4): 584-608. doi:10.1016/j.cedpsych.2007.08.002.

[7] Hulleman CS, Schrager SM, Bodmann SM, Harackiewicz JM A meta-analytic review of achievement goal measures: Different labels for the same constructs or different constructs with similar labels? Psychological Bulletin. 2010;136(3): 422–49. doi:10.1037/a0018947. 20438145

[8] Linnenbrink-Garcia L, Tyson DF, Patall EA When are achievement goal orientations beneficial for academic achievement? A closer look at main effects and moderating factors. Revue Internationale de Psychologie Sociale. 2008;21(1):19–70.

[9] Ranellucci J, Hall NC, Goetz T (2015). Achievement goals, emotions, learning and performance: A process model. Motivation Science. 2015;1(2): 98-120. doi:10.1037/mot0000014.

[10] Michou A, Vansteenkiste M, Mouratidis A, Lens W Enriching the hierarchical model of achievement motivation: Autonomous and controlling reasons underlying achievement goals. British Journal of Educational Psychology. 2014;84(4):650-66. doi:10.1111/bjep.12055. 25251866

[11] Pulfrey C, Buchs C, Butera F Why grades engender performance-avoidance goals: The mediating role of autonomous motivation. Journal of Educational Psychology. 2011;103(3):683. doi:10.1037/a0023911.

[12] Sideridis GD The regulation of affect, anxiety, and stressful arousal from adopting mastery-avoidance goal orientations. Stress and Health. 2008;24(1): 55-69. doi:10.1002/smi.1160.

[13] Vansteenkiste M, Smeets S, Lens W, Soenens B, Matos L, Deci EL Autonomous and controlled regulation of performance-approach goals: Their relations to perfectionism and educational outcomes. Motivation and Emotion. 2010;34(4): 333–53. doi:10.1007/s11031-010-9188-3.

[14] Ames C Classrooms: Goals, structures, and student motivation. Journal of educational psychology. 1992 Sep;84(3):261.

[15] Haynes TL, Daniels LM, Stupnisky RH, Perry RP, Hladkyj S The effect of attributional retraining on mastery and performance motivation among first-year college students. Basic and Applied Social Psychology. 2008 9 26;30(3):198-207.

[16] DeVoe P Learning in Medical School: Relationships Among Achievement Goals and Approaches to Learning in Three Classes of Medical Students [dissertation]. New Mexico, USA; The University of New Mexico; 2011 142 p.

[17] Chen HC, Cate O, O’Sullivan P, Boscardin C, Eidson-Ton WS, Basaviah P, Woehrle T, Teherani A Students’ goal orientations, perceptions of early clinical experiences and learning outcomes. Medical Education. 2016;50(2): 203–13. doi:10.1111/medu.1288. 26812999

[18] Bandura A Social foundations of thought and action: A social cognitive theory Englewood Cliffs: Prentice Hall 1986.

[19] Pekrun R, Vogl E, Muis KR, Sinatra GM Measuring emotions during epistemic activities: The epistemically-related emotion scales. Cognition and Emotion. 2016:1-9. doi:10.1080/02699931.2016.1204989. 27448030

[20] Schuwirth LW, Van der Vleuten CP Programmatic assessment: From assessment of learning to assessment for learning. Medical Teacher. 2011;33(6): 478-85. doi:10.3109/0142159X.2011.565828. 21609177

[21] Alkharusi H Effects of classroom assessment practices on students’ achievement goals. Educational Assessment. 2008;13(4):243-66.

[22] Huang C Achievement goals and self-efficacy: A meta-analysis. Educational Research Review. 2016 Nov 30;19:119-37.

[23] Huang C Achievement goals and achievement emotions: A meta-analysis. Educational Psychology Review. 2011 Sep 1;23(3):359.

[24] Richardson M, Abraham C, Bond R Psychological correlates of university students’ academic performance: a systematic review and meta-analysis. Psychological Bulletin. 2012;138(2):353. doi:10.1037/a0026838. 22352812

[25] Robbins SB, Lauver K, Le H, Davis D, Langley R, Carlstrom A Do psychosocial and study skill factors predict college outcomes? A meta-analysis. Psychological Bulletin. 2004;130:261–88. doi: 10.1037/0033-2909.130.2.261. 14979772

[26] Demirören M, Turan S, Öztuna D Medical students’ self-efficacy in problem-based learning and its relationship with self-regulated learning. Medical Education Online. 2016;21. doi:10.3402/meo.v21.30049. PMC4796725 26987386

[27] Bandura A Self-efficacy: The exercise of control Freeman; 1997.

[28] Pekrun R, Lichtenfeld S, Marsh HW, Murayama K, Goetz T Achievement emotions and academic performance: Longitudinal models of reciprocal effects. Child Development. 2017. 10.1111/cdev.12704 28176309

[29] Artino AR, La Rochelle JS, Durning SJ Second-year medical students’ motivational beliefs, emotions, and achievement. Medical Education. 2010;44(12):1203–12. doi:10.1111/j.1365-2923.2010.03712.x. 21091760

[30] Artino AR Jr, Holmboe ES, Durning SJ Control-value theory: using achievement emotions to improve understanding of motivation, learning, and performance in medical education: AMEE Guide No. 64. Medical Teacher. 2012;34(3):e148–60. doi: 10.3109/0142159X.2012.651515. 22364472

[31] Black P, Wiliam D Inside the black box: Raising standards through classroom assessment. Phi Delta Kappan. 2010;92(1):81-90.

[32] Lockyer J, Carraccio C, Chan MK, Hart D, Smee S, Touchie C, Holmboe ES, Frank JR; ICBME Collaborators Core principles of assessment in competency-based medical education. Med Teach. 2017;39(6):609-16. doi: 10.1080/0142159X.2017.1315082. 28598746

[33] Royal College of Physicians and Surgeons of Canada Competence by Design [Internet]. Available at: http://www.royalcollege.ca/rcsite/cbd/competence-by-design-cbd-e [Accessed April 10, 2018].

[34] Pekrun R, Cusack A, Murayama K, Elliot AJ, Thomas K The power of anticipated feedback: Effects on students’ achievement goals and achievement emotions. Learning and Instruction. 2014;29:115-24.

[35] Brookhart SM A theoretical framework for the role of classroom assessment in motivating student effort and achievement. Applied Measurement in Education. 1997;10(2):161-80.

[36] Sharma S, Sharma V, Sharma M, Awasthi B, Chaudhary S Formative assessment in postgraduate medical education-Perceptions of students and teachers. International Journal of Applied and Basic Medical Research. 2015;5(Suppl 1):S66. 26380215 10.4103/2229-516X.162282 PMC4552070

[37] Baranik LE, Barron KE, Finney SJ Measuring goal orientation in a work domain: Construct validity evidence for the 2 x 2 framework. Educational and Psychological Measurement. 2007;67(4):697-718. doi:10.1177/0013164406292090.

[38] Maehr ML, Zusho A Achievement goal theory. Handbook of motivation at school. 2009:77-104.

[39] Klassen RM, Durksen TL Weekly self-efficacy and work stress during the teaching practicum: A mixed methods study. Learning and Instruction. 2014;33:158-69. doi:10.1016/j.learninstruc.2014.05.003.

[40] Schwarzer R, Jerusalem M Generalized Self-Efficacy scale In: Weinman J, Wright S, Johnston M, (eds.) Measures in health psychology: A user’s portfolio. Causal and control beliefs. Windsor, United Kingdom: NFER-Nelson; 1995 p. 35-37.

[41] IBM Corp. Released 2015 IBM SPSS Statistics for Windows, Version 24.0 Armonk, NY: IBM Corp.

[42] Daniels LM From pre-service to practicing teacher: Considering the stability of personal and classroom mastery and performance goals. Educational Psychology. 2015;35(8):984-1005. doi:10.1080/01443410.2013.870329.

[43] Seo E, Lee Y, Patall EA Achievement Goals Predict Not Only Levels of Intrinsic Motivation but Also Its Stability. American Educational Research Association: San Antonio, Texas April, 2017.

[44] Linnenbrink-Garcia L, Patall EA, Pekrun R Adaptive motivation and emotion in education: Research and principles for instructional design. Policy Insights from the Behavioral and Brain Sciences. 2016;3(2):228-36.

[45] Perry RP, Chipperfield JG, Hladkyj S, Pekrun R, Hamm JM Attribution-based treatment interventions in some achievement settings In Motivational interventions 2014 (pp. 1-35). Emerald Group Publishing Limited.

